# Radiopharmaceutical Theranostics in Primary Adrenal Malignancies: A Surgeon’s Perspective

**DOI:** 10.3390/ph19050664

**Published:** 2026-04-24

**Authors:** Styliani Laskou, George Geropoulos, Petre Adrian Radu, Catalin Pirvu, Valeriu Surlin, Christoforos Kosmidis, Kyriakos Psarras, Stelian Pantea, Victor Strambu, Konstantinos Sapalidis

**Affiliations:** 1 3rd Surgical Department, AHEPA University Hospital, Aristotle University of Thessaloniki, 54636 Thessaloniki, Greece; 2 10th Department of Surgery, University of Medicine and Pharmacy “Carol Davila” Bucharest, 050474 Bucharest, Romania; 3 Department X, General Surgery II, Discipline of Surgical Emergencies, Victor Babes University of Medicine and Pharmacy Timisoara, E. Murgu Square, No. 2, 300041 Timisoara, Romania; 4 6th Department of Surgery, Emergency County Hospital Craiova Clinical, University of Medicine and Pharmacy of Craiova, 200642 Craiova, Romania

**Keywords:** adrenal malignancies, radiopharmaceutical theranostics, surgical triage, multidisciplinary management

## Abstract

Radiopharmaceutical Theranostics defines the combination of molecularly targeted imaging and therapy in two consecutive phases. Targeted theranostic approaches are most established for the management of advanced prostate, thyroid and hepatocellular cancer, as well as neuroendocrine tumors (NETs). Adrenal malignancies present a complex challenge, requiring highly specialized management. The two primary entities addressed by targeted radiotheranostics—pheochromocytoma/paraganglioma (PPGL) and adrenocortical cancer (ACC)—consist of fundamentally distinct molecular targets and, consequently, different radiopharmaceutical agents. While most existing literature focuses on nuclear medicine–driven perspectives, the implications of theranostic advances for surgical decision-making remain underexplored. This narrative review aims to integrate available clinical evidence with multidisciplinary practice considerations, in reshaping the role of surgery in adrenal malignancies.

## 1. Introduction

The term “theranostics” formulated in 1998 by John Funkhouser [1,2] refers to the seamless integration of a diagnostic biomarker and a therapeutic agent that share a specific target within tumor cells or their microenvironment [2,3]. In nuclear medicine, this paradigm utilizes the same vector (ligand) labeled with distinct radioisotopes—referred to as radiopharmaceutical—which serves both as a diagnostic radionuclide (e.g., positron emitter for PET) and a therapeutic radionuclide (e.g., beta-emitter) [4]. Radiopharmaceuticals have revolutionized the management of various cancers, maximizing the absorbed radiation dose within the tumor while sparing normal tissues, thereby achieving highly personalized and targeted therapy.

The first theranostic application lies back to 1946 when radioactive iodine (I-131) was successfully used in the management of thyroid diseases [5,6]. The principle applied is the natural ability of thyroid cells, mediated by the Sodium Iodide Symporter (NIS), to trap circulating radioiodine in both imaging and treatment [5]. Since then, radiopharmaceutical theranostics have been successfully implemented in several malignancies, most notably in prostate cancer with PSMA-targeted imaging and therapy, and in neuroendocrine tumors using somatostatin receptor–based approaches. These established models provide a conceptual and clinical framework for the development of theranostic strategies in adrenal malignancies.

Adrenal malignancies present a complex challenge, requiring highly specialized management. The two primary entities targetedby radiotheranostic strategies—pheochromocytoma (PHEO), arising from chromaffin cells of the adrenal medulla (with the term PPGL encompassing both PHEO and paragangliomas derived from extra-adrenal chromaffin tissue), and adrenocortical carcinoma (ACC)—display fundamentally distinct molecular profiles and therefore require different radiopharmaceutical agents. While surgical resection remains the standard of care for localized disease, a considerable proportion of patients present with advanced or metastatic tumors in which curative surgery is not feasible, underscoring the need for effective targeted molecular imaging and radiotherapeutic options. The evolving field of radiopharmaceutical theranostics enables a strategic understanding of tumor receptor status and systemic dose constraints. The molecular information derived from diagnostic theranostic scans is no longer solely the purview of the nuclear medicine physician; it is critical data that guides surgical triage, informs the extent of resection, and dictates the management of recurrent or metastatic disease. (Table 1). The contemporary approach emphasizes a multidisciplinary team model for optimal decision-making, integrating surgery with advanced molecular imaging and systemic therapies [7].

## 2. Methods

This study was conducted as a narrative review aimed at summarizing current evidence on radiopharmaceutical theranostics in primary adrenal malignancies, with a focus on clinical applicability and surgical decision-making. A literature search was performed using the PubMed/MEDLINE and Scopus databases to identify relevant studies published up to January 2026. The search strategy included combinations of the following keywords: “*adrenal malignancies*,” “*pheochromocytoma*,” “*paraganglioma*,” “*adrenocortical carcinoma*,” “*theranostics*,” “*MIBG*,” “*somatostatin receptor imaging*,” “*PRRT*,” “*FDG PET*,” “*CXCR4*,” “*PSMA*,” *and “FAP*.”

Articles were selected based on their relevance to theranostic applications in adrenal tumors. Priority was given to clinical studies, prospective and retrospective cohorts, meta-analyses, and key translational research addressing imaging and therapeutic radiopharmaceuticals. Review articles were also considered to provide contextual background and support interpretation of emerging data.

Given the narrative nature of this review, no formal systematic inclusion or exclusion criteria were applied. However, emphasis was placed on studies with clear clinical implications, particularly those informing multidisciplinary management and surgical decision-making.

Non-English publications and studies not directly related to adrenal tumor theranostics were excluded. The final selection of articles was based on relevance, methodological quality, and contribution to the evolving field of personalized oncologic care.

## 3. Theranostic Approaches for Pheochromocytoma and Paraganglioma (PPGL)

Pheochromocytomas and Paragangliomas (PPGLs) are rare neuroendocrine tumors, often developed on a background of predisposing genetic mutations. They present heterogeneous clinical and genomic portraits and, although the majority is benign, approximately 20% metastasize [8]. Given this complexity, the management of metastatic or inoperable PPGLs has shifted toward theranostics. Two principal targeted radionuclide therapies (TRTs) are currently established: Metaiodobenzylguanidine (MIBG) and Peptide Receptor Radionuclide Therapy (PRRT) using somatostatin analogues. The existence of two distinct theranostic pathways is dictated by the genotype-phenotype correlation within PPGLs. These tumors are generally categorized into three main molecular clusters:

- Cluster 1 consists of genes related to cell metabolism and the hypoxia response. It is often associated with Succinate Dehydrogenase (SDHx) or vin Hippel-Lindau (VHL) genes mutations. These tumors frequently exhibit high expression of Somatostatin Receptor Type 2 (SSTR2) but may show reduced expression of the Norepinephrine Transporter (NET) [8].
- Cluster 2 represents dysregulation of kinase signaling pathways. It is associated with RET, NF1, or MAX mutations. These tumors typically maintain high NET expression and catecholamine synthesis machinery [8].
- Cluster 3 (Wnt-altered/Non-Hypoxic): This cluster, characterized by somatic driver mutations like those in CSDE1 and MAML3 fusions, presents a significant therapeutic challenge [9]. These tumors typically exhibit low or absent expression of both the NET and SSTR targets, rendering them “double-negative” on conventional theranostic imaging.

The recognition of these three molecular subgroups (Clusters) is fundamental. However, given that Cluster 3 currently does not respond to established approaches, clinical practice remains focused on the receptors expressed in Clusters 1 and 2.

### 3.1. The Norepinephrine Transporter Pathway: Metaiodobenzylguanidine (MIBG)

MIBG is a structural iodinated guanethidine analog of norepinephrine that is actively taken up by chromaffin tissue via the NET and stored in vesicular granules facilitated by vesicular monoamine transporters. ^123/131^I-MIBG represents a well-established theranostic pair. According to the European Association of Nuclear Medicine, ^123^I-MIBG is superior in terms of PPGL diagnosis since it demonstrates shorter half-life and better image quality but lower radiation burden [10]. While early studies reported high sensitivity for non-metastatic adrenal pheochromocytomas (approximately 83–100%) with excellent specificity, larger cohorts including extra-adrenal and hereditary cases demonstrate significantly lower sensitivity (approximately 52–75% for mixed PPGL populations). In metastatic disease, sensitivity may range from 56% to 83%, and per-lesion detection can be below 60% [11]. Importantly, tumors associated with SDHx mutations, particularly SDHB, show markedly reduced ^123^I-MIBG uptake, with reported sensitivities often below 50%, reflecting diminished expression of the norepinephrine transporter in these genotypes [12]. Although ^123^I-MIBG scintigraphy has its limitations, it is crucial as a precursor to 131IMIBG therapy.

^131^I-MIBG consists of a treatment option for unresectable or metastatic diseases PPGL in whom the diagnostic scan is positive done either with ^123^I/^131^I-MIBG [13].

Historically, treatment has been performed using conventional low–specific-activity (LSA) ^131^I-MIBG, defined as a formulation in which the majority of administered MIBG molecules are non-radioactive (“cold” carrier), with only a small fraction labeled with ^131^I. This excess of unlabeled MIBG results in competitive inhibition at the norepinephrine transporter (NET), potentially limiting tumor uptake and reducing therapeutic radiation delivery.

Clinical studies using LSA ^131^I-MIBG have demonstrated symptomatic improvement and biochemical responses, with objective tumor response rates typically ranging from 20% to 30% and disease control rates exceeding 50% in selected series. However, therapeutic efficacy may be suboptimal in patients with high tumor burden or heterogeneous NET expression [14,15,16].

High–specific-activity (HSA) ^131^I-MIBG was developed to overcome this limitation. As a carrier-free formulation, HSA MIBG minimizes pharmacologic competition and enables higher intracellular radiation delivery. Prospective trials have demonstrated improved clinical outcomes, including durable reductions in catecholamine levels, improved blood pressure control, and sustained disease stabilization. Importantly, HSA ^131^I-MIBG has been associated with a more predictable toxicity profile, with myelosuppression representing the main dose-limiting effect [17,18,19].

Patient selection remains critical, as both LSA and HSA therapies require demonstrable MIBG-avid disease on diagnostic imaging. HSA ^131^I-MIBG is generally preferred in patients with advanced, progressive disease and adequate bone marrow reserve, whereas lack of tracer uptake or dedifferentiated tumor biology limits its applicability [17,18,19].

### 3.2. The Somatostatin Receptor Pathway: PPRT

Somatostatin receptors (SSTRs) are G-protein–coupled receptors with five known subtypes (SSTR1–5), of which SSTR2 and SSTR3 is the most consistently and highly expressed in PPGL [20]. SSTR expression is observed on tumor cell membranes and reflects neuroendocrine differentiation, providing the molecular basis for somatostatin analogue–based imaging and therapy. Early studies employing ^111^In-pentetreotide (Octreoscan^®^) demonstrated improved sensitivity over MIBG scintigraphy for the detection of metastatic paragangliomas and head and neck paragangliomas, although its performance was limited in primary tumors [21,22]. In settings where PET tracers are not available, Tc-99m–labeled somatostatin analogues (e.g., Tc-99m-HYNIC-TOC) represent a practical and accessible SPECT-based alternative, although with lower sensitivity and spatial resolution compared to PET imaging. The advent of positron emission tomography (PET)–based SSTR imaging has largely replaced single-photon techniques due to superior spatial resolution, image quality, and quantitative capabilities. ^68^Ga-labeled DOTA-conjugated somatostatin analogues—including ^68^Ga-DOTATATE, ^68^Ga-DOTATOC, and ^68^Ga-DOTANOC—have been extensively validated for PPGL detection and, despite differences in affinity for SSTR subtypes, all exhibit high sensitivity for SSTR2, the predominant receptor expressed in these tumors [20]. Across multiple studies, ^68^Ga-DOTA-SSA PET has demonstrated overall sensitivities ranging from 80% to 100%, outperforming other functional imaging modalities such as ^18^F-FDOPA, ^18^F-FDA, ^18^F-FDG, and ^123^/^131^I-MIBG, particularly in metastatic, extra-adrenal, multifocal, and SDHB-mutated disease, where reported sensitivities often exceed 90% [23,24,25,26,27,28,29].

Nevertheless, diagnostic performance may vary depending on tumor biology, as highlighted by reduced detection rates in specific subgroups, underscoring the complementary role of alternative tracers. Owing to its robust diagnostic accuracy and theranostic relevance, ^68^Ga-DOTA-SSA PET has received regulatory approval and is recommended by international guidelines as the first-line imaging modality in selected PPGL scenarios, while simultaneously serving as a critical tool for identifying candidates suitable for somatostatin receptor–targeted radionuclide therapy [10].

PRRT using radiolabeled somatostatin analogues—most commonly ^177^Lu-DOTATATE and ^90^Y-DOTATOC—has been increasingly investigated in this rare tumor population. Evidence supporting PRRT efficacy in PPGL is largely derived from retrospective series and small prospective cohorts; nevertheless, a recent meta-analysis encompassing 12 studies and 201 patients demonstrated an objective response rate of approximately 25%, with disease control achieved in 84% of cases [30]. Biochemical and symptomatic responses were observed in 64% and 61% of patients, respectively, while mean progression-free and overall survival were reported as 37.1 and 54.5 months [30]. Importantly, baseline uptake intensity on somatostatin receptor imaging has been consistently associated with therapeutic response and survival outcomes, with higher SUVmax values predicting improved tumor control and longer progression-free survival, supporting the theranostic principle of imaging-guided treatment selection [31]. Additional factors influencing PRRT efficacy include tumor differentiation, Ki-67 index, genetic background, disease distribution, and treatment sequencing, with several studies suggesting particularly favorable outcomes in SDHx-mutated paragangliomas and parasympathetic tumors [32,33]. Although pheochromocytomas appear to derive less benefit than paragangliomas—possibly due to lower or heterogeneous SSTR expression—the overall body of evidence supports PRRT as an effective and biologically sound therapeutic strategy in appropriately selected SSTR-positive PPGL patients, despite heterogeneity across studies and the need for further prospective validation [26].

In addition to receptor-targeted imaging, ^18^F-FDG PET plays a critical complementary role, particularly in aggressive or dedifferentiated PPGLs. Increased FDG uptake is commonly observed in tumors associated with SDHx mutations and is linked to higher metastatic potential and poorer prognosis. In such cases, FDG PET may outperform other tracers and is often integrated into a multi-tracer imaging strategy to better characterize tumor biology. The combined use of SSTR imaging, MIBG, and FDG PET reflects a shift toward personalized imaging algorithms, where tracer selection is guided by genotype, tumor differentiation, and clinical behavior.

It should be noted that the majority of evidence supporting these approaches is derived from retrospective analyses, small prospective cohorts, and non-randomized studies, with a lack of large randomized controlled trials. In this context, “selected patients” refers to individuals with demonstrable tracer uptake on diagnostic imaging (e.g., MIBG-avid or SSTR-positive disease), adequate organ function (particularly bone marrow reserve), favorable performance status, and disease characteristics such as well-differentiated or receptor-expressing tumors.

## 4. Theranostic Approaches for Adrenocortical Carcinoma (ACC)

ACC with an annual incidence of 2 cases per 1 million people represents a rare tumor from the cortex of the adrenal gland [34]. In terms of management a multidisciplinary approach by endocrine surgeon, endocrinologist and oncologist is required due to the adrenal localization, the aggressive biology and the possible hormone-excess syndrome presentation.

Current guidelines suggest that surgical resection is the only curative option for the management of localized disease without evidence of metastasis. Local recurrence rate ranges from 19% to 34%, depending on tumor stage. Surgery is also recommended for recurrent disease with low tumor burden if R0 resection is achievable. Adjuvant therapy is mandatory, while neoadjuvant therapy with mitotane may be considered for patients with borderline resectable tumors.

Mitotane is the cornerstone of adjuvant therapy. Regimens combining mitotane with cisplatin-based chemotherapyplus mitotane are recommended (e.g., Ki67 ≥ 30%, large tumor thrombus) but lack strong evidence, while the role of radiotherapy remains unclear but may benefit patients with stage III node-negative ACC [35,36]. For advanced adrenocortical carcinoma treatment must be considered palliative improving quality of life thus minimizing adverse effects from antineoplastic therapies [37]. Mitotane in combination with the conventional cytotoxic FIRM-ACT protocol (EDP-M: etoposide, doxorubicin, cisplatin, and mitotane) is the standard of care for such patients. Other regimens, such as gemcitabine-based combinations, have shown limited efficacy [38], while targeted therapies, such as Tyrosine Kinase Inhibitors like sunitinib and cabozantinib and IGF-2 Axis Inhibitors like linsitinib, have been studied with modest results. Immunotherapy with pembrolizumab has shown some promise with an overall response rate (ORR) of 23% in a phase II trial [39]. Nivolumab and avelumab have also shown limited efficacy [40]. Combination therapies, such as nivolumab with ipilimumab, are under investigation [37].

While these systemic and locoregional therapies constitute the current standard of care, radiotheranostic approaches should be viewed as complementary rather than competing strategies. The integration of radiopharmaceutical therapies into existing treatment algorithms requires careful multidisciplinary assessment.

Patient selection is even more restrictive and depends on sufficient target expression (e.g., CYP11B positivity, CXCR4 expression), limited therapeutic alternatives, and advanced or refractory disease status. ^18^F-FDG PET is widely used in staging and restaging, reflecting tumor metabolic activity and aggressiveness, and may provide prognostic information complementary to receptor-targeted imaging.

It should be emphasized that, unlike PPGL, theranostic applications in ACC remain largely investigational, with no established role in standard clinical practice and limited evidence derived primarily from early-phase studies, small cohorts, and case reports, with no randomized trials available to date. Strategies focus on highly specific pathways: enzyme-targeted approaches utilizing agents like IMAZA to exploit 11β-hydroxylase expression, and CXCR4-directed imaging and therapy. Although secondary targets like somatostatin receptors (SSTR) and fibroblast activation protein (FAP) remain in the early stages of investigation—with PRRT data currently limited to isolated case series—these modalities offer a critical “last-resort” option for refractory disease [41]. Ultimately, the transition of these tracers from promising experimental tools to standard care depends on ongoing clinical trials and rigorous patient selection facilitated by high molecular imaging uptake, ensuring that the radioactive payload is delivered only to those likely to benefit [42].

Research efforts have primarily focused on the CYP11B enzyme family, with ^123/131^IMAZA and its predecessor, ^123/131^IMTO, emerging as the most significant developments in this field. These tracers function by selectively binding to CYP11B1 (11β-hydroxylase) and CYP11B2 (aldosterone synthase), enzymes that are expressed exclusively within the adrenal cortex and are known to accumulate significantly in ACC tissues [43]. The clinical viability of this approach was evidenced in a small-scale study of eleven patients with advanced ACC, where treatment with ^131^IMTO demonstrated both high tolerability and meaningful therapeutic impact [44]. In this cohort, 54% of patients achieved disease control for at least five months, with a median progression-free survival of 14 months. Ultimately, the efficacy of such radionuclide therapies is dictated by a combination of factors, including the level of intratumoral enzyme expression, the inherent radiosensitivity of the malignant tissue, and the duration of radiotracer retention within the tumor lesions [44]. However, given the rapid metabolic inactivation of [^123^I] IMTO in vivo, a novel molecule with greater metabolic stability could enhance and extend target binding, which would subsequently lead to higher tumor dosages. [^123^I]IMAZA for imaging and [^131^I]IMAZA for therapy represent the promosing therapeutic pair. Actually, in a prospective series of 69 metastatic, unresectable ACC patients, 38.5% had sufficient [^123^I]IMAZA uptake; 13 received [^131^I]IMAZA (median 25.7 GBq) [45]. [^131^I]IMAZA demonstrated promising clinical activity, achieving disease stabilization in five patients with a median progression-free survival of 14.3 months and a median overall survival of 14.1 months. The therapy was well tolerated with no reported CTCAE grade 3 or higher toxicities, and notably resulted in partial tumor shrinkage of up to −26% in two patients [45]. Michalski et al. and Kiesewetter et al. as well, also identify the {^123/131^}I]IMAZA”theranostic twin” as the primary adrenal-enzyme–based radiopharmaceutical with repeated human use [37,42]. While clinical evaluations demonstrate a “promising effect in selected patients” the platform is not broadly applicable across all ACC phenotypes. Its reach is constrained by the significant biological heterogeneity of the disease; intense tracer uptake is restricted to the more differentiated, enzyme-positive subgroup (approximately 38.5% of advanced cases), whereas the most aggressive, undifferentiated tumors often fail to express the necessary molecular targets [37,42,45]. A radiopharmaceutical with a broader application range for ACC remains a major unmet need.

The necessity of treating undifferentiated and CYP11B-negative tumors has spurred investigation into non-cortical targets established in other cancer types. The C-X-C motif chemokine receptor 4 (CXCR4) represents a molecular target that could potentially enhance the therapeutic regimen in a subgroup of ACC patients. High expression of CXCR4 can be visualized using the PET tracer Ga-68 Pentixafor, providing a potential avenue for targeted radiotherapy [42,46]. Evidence is limited to small series and case reports; thus therapeutic efficacy is not yet systematically quantified. CXCR4-radioligand therapy could be proposed as a last-line option in end-stage disease [45,46].

Prostate-specific membrane antigen (PSMA) has also emerged as a potential theranostic target in ACC. Although originally characterized in prostate cancer, PSMA expression has been demonstrated in the neovasculature of several solid tumors, including ACC. Crowley et al. showed that PSMA is expressed in the tumor microvasculature of ACC and proposed that it may represent a potential anti-angiogenic therapeutic target, supporting the biological rationale for PSMA-directed imaging and therapy in this disease [47]. More recently, clinical imaging observations have further explored this concept. Hahner et al. reported the feasibility of PSMA-directed PET/CT in ACC and highlighted the growing interest in theranostic approaches targeting cell-surface receptors such as CXCR4 and PSMA. Nevertheless, clinical experience remains limited, and uptake in some metastatic lesions appears modest, suggesting that PSMA may be less sensitive than CXCR4-based imaging in certain clinical scenarios [48].

Beyond tumor-cell and neovasculature-directed targets, theranostic strategies directed at the tumor microenvironment are increasingly being investigated. Fibroblast activation protein (FAP), expressed by cancer-associated fibroblasts, represents a promising stromal target that may allow imaging and therapy of ACC independent of tumor-cell receptor heterogeneity. In a recent clinical report, Chopra et al. explored FAP-targeted radiopharmaceuticals in ACC and demonstrated the feasibility of FAP-directed imaging, highlighting the potential role of FAP-based agents as an additional theranostic avenue. Although these early findings are encouraging, current evidence is limited to preliminary clinical experience, and larger studies are required to clarify the diagnostic performance and therapeutic value of FAP-targeted approaches in ACC [49].

Somatostatin receptor–targeted imaging and peptide receptor radionuclide therapy (PRRT) represent another potential theranostic avenue in ACC, although evidence remains limited and heterogeneous. Expression of SSTRs has been demonstrated in a subset of ACCs, and functional imaging with radiolabeled somatostatin analogues (e.g., ^68^Ga-DOTATOC or DOTATATE) can identify patients with sufficient receptor expression for targeted approaches. However, uptake is variable, and only a minority of patients appear to be suitable candidates for PRRT, reflecting the biological heterogeneity of ACC [50].

Clinical experience with SSTR-directed therapy in ACC remains limited to small series and case-based evidence. Reviews of ACC management emphasize that radionuclide-based or receptor-targeted treatments, including PRRT, should currently be regarded as investigational strategies or options in highly selected patients, generally in the setting of advanced or refractory disease [37].

Overall, these approaches should currently be regarded as exploratory and limited to selected patients, typically in the context of advanced, refractory disease or clinical trials, pending further validation.

## 5. The Interface of Surgery and Radiotheranostics: An Endocrine Surgeon’s Perspective

For the endocrine surgeon, adrenal radiotheranostics contribute significantly across three clinical pillars: enabling initial triage, guiding the extent of resection, and individualizing post-operative management. While the majority of adrenal lesions are adequately managed using CT and MRI, functional imaging provides a metabolic map that goes beyond anatomy. Radiotheranostics allow for the precise characterization of benign versus malignant tumors and the early detection of occult metastatic disease. This “triage” is critical; it dictates the clinical priority and the nature of the intervention. For instance, identifying a high SSTR expression in a PPGL patient via Ga-DOTATATE not only confirms the diagnosis but immediately identifies the patient as a candidate for PRRT should surgery not be curative.

The surgical strategy is directly informed by the “intensity” and distribution of the radiotracer. For ACC amenable to excision, an R0 resection remains the gold standard. To achieve this in locally advanced cases, an aggressive en bloc surgical approach is mandatory. This often necessitates the multivisceral resection of adjacent structures (e.g., kidney, spleen, or liver segments) to ensure the tumor remains encapsulated and to prevent intraoperative rupture or spillage—events that drastically worsen prognosis [36].

In the context of PPGL, radiotheranostics help the surgeon distinguish between a solitary lesion and multifocal disease, which is common in hereditary syndromes. This ensures that the surgical plan is sufficiently broad to address all hypersecreting tissue, while also being conservative enough to preserve organ function where possible. Furthermore, locoregional lymphadenectomy guided by preoperative functional “hotspots” is essential for precise pathological staging and improved long-term oncologic outcomes [36].

The “theranostic” bridge is most evident in the post-operative phase. If R0 resection is achieved, radiotheranostics serve as a highly sensitive surveillance tool for early recurrence, often detecting biochemical relapses before they are visible on structural imaging. In cases of advanced ACC not amenable to complete resection, or metastatic PPGL where surgery is palliative (focusing on cytoreduction to mitigate catecholamine-induced symptoms), radiotheranostics transition from “diagnosis” to “therapy.” For example, patients whose tumors showed high uptake on preoperative scans can be seamlessly transitioned to targeted radionuclide therapies, such as ^131^I-MIBG or ^177^Lu-DOTATATE. This personalized approach ensures that systemic treatment is biologically targeted to the specific characteristics of the patient’s tumor, optimizing the management of the remaining tumor burden and improving the quality of life.

Despite promising clinical results, several practical barriers limit the widespread implementation of radiotheranostics in adrenal malignancies. These include the limited availability of specific radiopharmaceuticals, particularly in centers without access to PET tracers or radionuclide therapy facilities, as well as the need for specialized infrastructure and multidisciplinary expertise for safe delivery of treatments such as PRRT or ^131^I-MIBG. Additionally, regulatory approval and reimbursement policies vary significantly across countries, which may restrict patient access to these therapies. Addressing these challenges is essential for translating theranostic advances into routine clinical practice.

## 6. Conclusions

Radiopharmaceutical theranostics represent a paradigm shift in the management of primary adrenal malignancies, evolving from purely diagnostic tools to integral components of the surgical armamentarium. By bridging the gap between molecular biology and clinical intervention, this approach allows for a level of precision previously unattainable in endocrine oncology. Ultimately, the transition from “anatomy-based” to “biology-based” surgery is facilitated by the Multidisciplinary Team (MDT). The integration of nuclear medicine and surgical expertise ensures that each patient receives a truly personalized treatment plan. Future research should focus on expanding the tracer library to cover “double-negative” or undifferentiated phenotypes, ensuring that the radioactive payload can be delivered to even the most aggressive adrenal malignancies.

## Figures and Tables

**Table 1:** Current Theranostic Approaches in Adrenal Malignancies.

Target	Imaging Modality	Therapeutic Agent	Indication	Level of Evidence	Key Limitations
NET (MIBG)	^123^I-MIBG	^131^I-MIBG	PPGL	Phase II	Requires sufficient uptake; myelotoxicity
SSTR	^68^Ga-DOTATATE	^177^Lu-DOTATATE	PPGL	Meta-analyses	Heterogeneous receptor expression
CYP11B	^123^I-IMAZA	^131^I-IMAZA	ACC	Early-phase studies	Limited to enzyme-positive tumors
CXCR4	^68^Ga-Pentixafor	Experimental	ACC	Case series	Limited validation
PSMA	PSMA PET	Experimental	ACC	Case reports	Variable uptake
FAP	FAPI PET	Experimental	ACC	Early clinical data	Very limited evidence

Abbreviations: PPGL, pheochromocytoma and paraganglioma; ACC, adrenocortical carcinoma; NET, norepinephrine transporter; MIBG, metaiodobenzylguanidine; SSTR, somatostatin receptor; PRRT, peptide receptor radionuclide therapy; PSMA, prostate-specific membrane antigen; FAP, fibroblast activation protein.

## Data Availability

No new data were created or analyzed in this study.
